# Exploring multidrug resistance patterns in community-acquired *Escherichia coli* urinary tract infections with machine learning

**DOI:** 10.1128/aac.00422-25

**Published:** 2025-10-31

**Authors:** Elise Hodbert, Olivier Lemenand, Sonia Thibaut, Thomas Coeffic, David Boutoille, Stephane Corvec, Gabriel Birgand, Laura Temime, on behalf of the French Clinical Laboratories Nationwide Network

**Affiliations:** 1 Cibles et médicaments des infections et de l’immunité, IICiMed, Nantes Université 27045, Nantes, France; 2 Laboratoire Modélisation, Epidémiologie et Surveillance des Risques Sanitaires (MESuRS), Conservatoire National des Arts et Métiers 27054, Paris, France; 3 Centre d’Appui à la Prévention des Infections Associées aux Soins des Pays de la Loire, Centre Hospitalier Universitaire (CHU) - Le Tourville 26922, Nantes, France; 4 French National Surveillance System of Antimicrobial Resistance in Primary Care and Nursing Homes, PRIMO, CHU - Le Tourville 26922, Nantes, Pays de la Loire, France; 5 Department of Infectious Diseases, University Hospital of Nantes and Center d’Investigation Clinique 1413, Institut National de la Santé et de la Recherche Médicale, Nantes, CHU Nantes 26922 , Nantes, France; 6 Service de Bactériologie, CHU Nantes 26922 , Nantes, France; 7 INSERM, Immunology and New Concepts in ImmunoTherapy, INCIT, UMR 1302, Nantes Université 27045, Nantes, France; 8 National Institute for Health Research Health Protection Research Unit in Healthcare Associated Infections and Antimicrobial Resistance, Imperial College London, London, United Kingdom; 9 Unité PACRI, Institut Pasteur, Conservatoire National des Arts et Métiers 27054, Paris, France; Columbia University Irving Medical Center, New York, New York, USA

**Keywords:** machine learning, community-acquired *E. coli* urinary tract infection, multiresistance pattern, antibiotic resistance, network analysis

## Abstract

While associations of antibiotic resistance traits are not random in multidrug-resistant (MDR) bacteria, clinically relevant resistance patterns remain underexplored. This study used association-set mining to explore resistance associations within *E. coli* isolates from community-acquired urinary tract infection isolates collected from 2018 to 2022 by France’s national surveillance system. Association-set mining was applied separately to extended-spectrum beta-lactamase-producing *E. coli* (ESBL-EC) and non-ESBL-EC. MDR patterns with expected support (reflecting pattern frequency) and conditional lift (reflecting association strength) higher than expected by chance (*P*-value ≤ 0.05) were used to construct resistance association networks and analyzed according to time, age, and gender. The number of isolates increased from 360,287 in 2018 to 629,017 in 2022. More MDR patterns were selected in ESBL-EC than non-ESBL-EC (2022: 1770 vs 93 patterns), with higher respective network densities (2022: 0.301 vs 0.100). Fluoroquinolone, third-generation cephalosporin, and penicillin resistance were strongly associated in ESBL-EC. Median network densities increased from 2018 to 2022 in both ESBL-EC (0.238–0.301, *P*-value = 0.06, Pearson test) and non-ESBL-EC (0.074–0.100, *P*-value = 0.04). Across all years, median densities were higher in men than in women (ESBL-EC 2022: 0.305 vs 0.271; non-ESBL-EC: 0.128 vs 0.094) and higher in individuals over 65 than under 65 (ESBL-EC: 0.289 vs 0.275; non-ESBL-EC: 0.103 vs 0.088). These findings highlight temporal, age-specific, and gender-specific variations in resistance patterns, underscoring the potential of machine learning to understand them and inform antibiotic strategies.

## INTRODUCTION

Antimicrobial resistance (AMR) is an increasing public health issue, notably due to the extensive use of antibiotics and increasing global mobility. Infections caused by multidrug-resistant bacteria (MDR) are challenging to treat, resulting in increased mortality, prolonged hospital stays, and higher healthcare costs (1). Initially regarded as a hospital-specific issue, AMR is now recognized as a major problem in community settings and a one-health issue (2). In 2021, AMR-associated infections were directly responsible for approximately 1.14 million deaths globally (3). Multiple studies forecast an increase in resistance to available antibiotics, which could cause more than 8 million deaths in 2050 (3). The global burden of AMR is driven by a few leading microorganisms, among which resistant *E. coli* infections caused the highest number of deaths in 2019 (4). In the community, *E. coli* accounts for 75%–90% of uncomplicated urinary tract infection (UTI) isolates (5). The proportion of extended-spectrum beta-lactamase-producing and MDR community-acquired *E. coli* UTI is rising, increasing from 2.8% in 2021 to 3.4% in 2023 (6).

MDR does not result from individual drug resistances occurring together by chance, as documented in multiple bacteria (7). Beyond selection pressure, several biological mechanisms are known to contribute to MDR emergence and spread *in E. coli*. Non-specific mechanisms, like efflux pumps, typically confer resistance to a wide range of antibiotics, whereas specific mechanisms, on the other hand, include notably the production of extended-spectrum-beta-lactamases and chromosomal point mutations conferring resistance to fluoroquinolone (7). MDR spreads through various pathways, most notably horizontal gene transfer (8).

However, MDR patterns and their variations across time, space, and subpopulations are not fully understood. A detailed analysis of these patterns could provide insight for optimized stewardship strategies of current antibiotics, by helping treat MDR infections with antibiotics carrying few co-resistances. Understanding temporal dynamics in multiresistance patterns may also help to better define guidelines for narrowing treatment spectrum and better preserving antibiotic efficacy over time. This is a key issue in the context of rising AMR and a limited antibiotic development pipeline.

Here, we use association-set mining, a machine-learning approach, to study MDR patterns in community-acquired *E. coli* UTIs. This method has proven effective in identifying patterns of antibiotic resistance traits in chicken-associated *E. coli*, human-associated *S. aureus*, and cattle-associated *S. enterica* (9–11). We analyze data collected over 5 years (2018–2022) by the French nationwide surveillance system of clinical laboratories, PRIMO (Surveillance and Prevention of Antibiotic Resistance in Primary Care and Nursing Homes) (12). We create graphical networks from the patterns detected by the algorithm in order to select resistance associations of interest. We then explore how these resistance associations have changed over time and perform gender- and age-stratified analyses, highlighting temporal trends and differences across subpopulations.

## RESULTS

### Data description

The PRIMO data sets include antibiotic susceptibility testing (AST) results, patient’s age, gender, and administrative region. We restricted the analyses to data collected from *E. coli* UTIs in community laboratories between 1 January 2018 and 31 December 2022. The number of isolates increased over time, from 360,287 in 2018 to 628,993 in 2022 (Table 1; *P*-value = 0.01, Pearson trend test). The proportion of extended-spectrum beta-lactamases producing *E. coli* (ESBL-EC), either alone or combined with another phenotype, was comprised between 2.8% and 3.0%, with no increasing or decreasing trend across the years (*P*-value = 0.79, Pearson trend test). Throughout all years, between 84.0% and 84.5% of isolates came from women. Among women, the proportion of isolates coming from individuals aged 65 and older ranged from 45.6% to 49.0% (*P* = 0.008, Pearson trend test), whereas among men, this proportion varied between 57.7% and 62.4% (*P* = 0.002, Pearson trend test). The isolates came from all regions of metropolitan France, with varying proportions across the years (Fig. S1).

**TABLE 1:** Summary of PRIMO data sets for *E. coli* UTI samples from 2018 to 2022

	2018	2019	2020	2021	2022
Total number of isolates	360,287	450,820	450,135	571,126	628,993
ESBL	10,150 (2.8%)	13,559 (3.0%)	13,265 (2.9%)	16,006 (2.8%)	18,663 (3.0%)
Non-ESBL	350,137 (97.2%)	437,261 (97.0%)	436,870 (97.1%)	555,120 (97.2%)	610,330 (97.0%)
Women	276,220 (84.5%)	356,002 (84.4%)	361,758 (84.0%)	457,789 (84.3%)	520,081 (84.1%)
65*+* years old	125,575 (45.6%)	163,453 (46.1%)	173,029 (47.9%)	219,423 (48.0%)	254,994 (49.0%)
Men	50,706 (15.5%)	66,000 (15.6%)	68,730 (16.0%)	85,444 (16.7%)	98,055 (15.9%)
65*+* years old	29,088 (57.7%)	38,543 (58.6%)	41,484 (60.5%)	51,873 (60.8%)	61,063 (62.4%)

### Individual antibiotic resistance prevalence in ESBL-EC and non-ESBL-EC

We selected antibiotics tested in the AST panels used in routine by participating clinical laboratories, with a cut-off of at least 10% of isolates tested across all years to avoid potential selection bias of antibiotics tested only on resistant strains and antibiotics rarely used in routine, which resulted in the selection of 27 antibiotics (Table S2).

**Fig 1:**
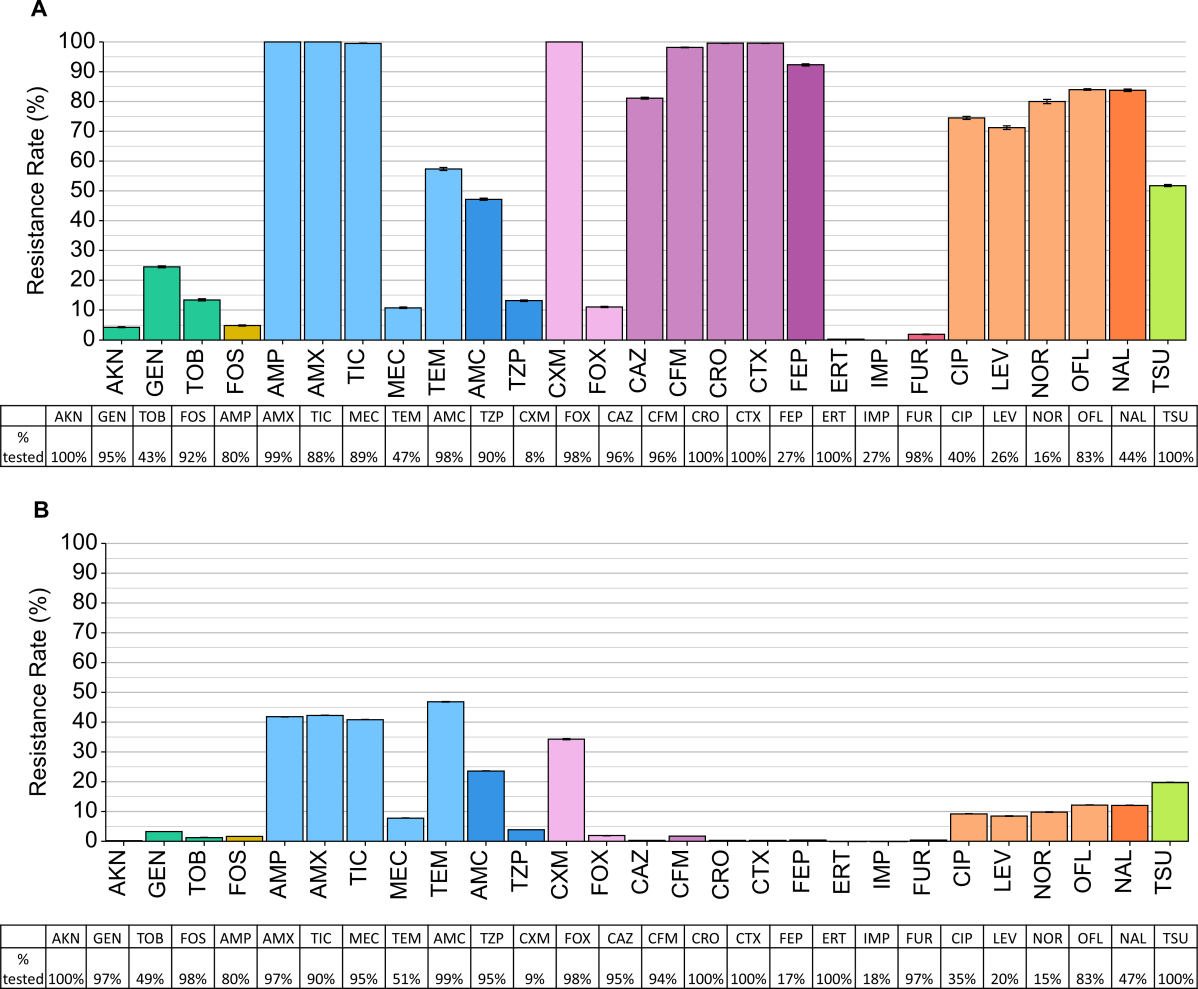
Resistance prevalence and AST frequency for all antibiotics in 2022 in (**A**) ESBL-EC and (**B**) non-ESBL-EC. See Table S2 for a complete list of antibiotics with their full names.

Regarding penicillins, nearly all ESBL-EC were resistant to ampicillin, amoxicillin, and ticarcillin (Fig. 1A). Most ESBL-EC was resistant to second- and third-generation cephalosporins, including cefuroxime (100%, 1,535/1,535 tested in 2022), ceftriaxone (99.6%, 18,592/18,663 tested) and cefotaxime (99.6%, 18,592/18,663 tested). For fluoroquinolones, between 70% and 85% of ESBL-EC were resistant to ciprofloxacin, levofloxacin, norfloxacin, and ofloxacin. However, most ESBL-EC were sensitive to first-line antibiotics, including fosfomycin (4.9%, 834/17,180 tested), nitrofurantoin (1.9%, 348/18,259 tested), mecillinam (10.8%, 1,800/16,684 tested), and cefoxitin (11.1%, 2,035/18,380 tested).

Non-ESBL-EC was often resistant to penicillins, including ampicillin (41.8%, 203,547/486,592 tested), amoxicillin (42.3%, 251,107/593,743 tested), and ticarcillin (40.9%, 223,404/546,477 tested; Fig. 1B). Less than 1% of non-ESBL-EC were resistant to third-generation cephalosporins including ceftazidime, cefixime, ceftriaxone, and cefotaxime. For fluoroquinolones, between 5% and 15% of isolates were resistant to ciprofloxacin, levofloxacin, norfloxacin, and ofloxacin. Similar individual antibiotic resistance prevalences were found from 2018 to 2021 (Fig. S2).

### Independence of individual resistance traits

**Fig 2:**
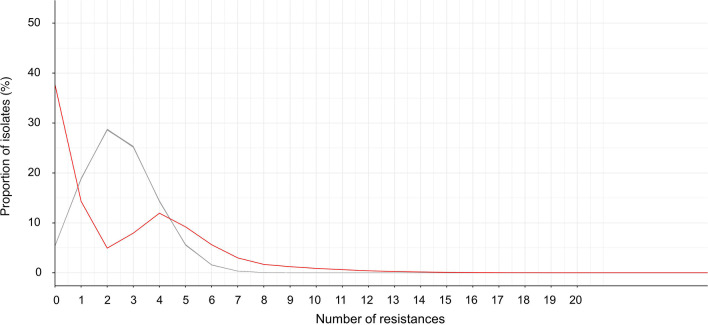
Distribution of the number of resistances per isolate in 2022 for all isolates. The red line represents the observed data set, and the gray line represents the simulated data sets.

We assessed the independence of individual resistance traits by simulating 100 data sets under the hypothesis that all resistance traits are mutually independent. We then compared the distribution of resistance counts per isolate between the observed and simulated data. In 2022, the observed distribution differed significantly from the distribution simulated under the hypothesis of independence (*P*-value = 6.81.10^−3^, Kolmogorov-Smirnov test), suggesting that individual resistances were not independent (Fig. 2). In the observed data set, 39% of isolates were pansusceptible, compared to 9% in the simulated data set. Similar results were obtained when comparing the distribution of the number of resistances per isolate in observed and simulated data from 2018 to 2021 (Fig. S3). We also compared the distributions separately for ESBL-EC and non-ESBL-EC from 2018 to 2022 (Fig. S4), underlining that the difference was mostly driven by non-ESBL-EC.

### Multiresistance pattern selection

We identified MDR patterns separately for each year and phenotype data set, using association-set mining with the *Apriori* algorithm (10, 13). We first explored the data sets to select all patterns present with a given frequency, and in a second step, we selected patterns for which two important metrics, expected support (eSup) and conditional lift (cLift), were significantly higher than expected by chance.

For ESBL-EC, the *Apriori* algorithm initially identified 52,824 patterns in 2018 and 123,116 patterns in 2022, with an increasing trend over the years (*P*-value = 0.01, Pearson test) (Table 2). For non-ESBL-EC, the algorithm initially generated 2,529 patterns in 2018 and 4,515 patterns in 2022, with a non-significant increasing trend (*P*-value = 0.06, Pearson test).

**TABLE 2:** Number of multiresistance patterns selected by association-set mining for each year and phenotype subset^*a*^

Phenotype	Year	No. of patterns initially generated by the *Apriori* algorithm	eSup cut-off value	cLift cut-off value	No. of patterns selected after pruning
ESBL	2018	52,736	0.257	1.076	483
	2019	86,454	0.236	1.070	1,234
	2020	106,625	0.242	1.073	1,268
	2021	115,425	0.262	1.065	1,921
	2022	123,116	0.271	1.058	1,770
Non-ESBL	2018	2,527	0.030	1.056	59
	2019	3,936	0.029	1.051	82
	2020	3,756	0.030	1.052	75
	2021	4,945	0.031	1.045	88
	2022	4,502	0.034	1.041	93

^*a*^ For example, in 2018 ESBL-EC, 52,736 patterns were identified. Cut-off values were set at eSup ≥ 0.257 and cLift ≥ 1.076, meaning that a pattern was retained if it appeared in at least 25.7% of isolates and occurred 7.6% more frequently than expected under the assumption of independence between resistance traits. After pruning, 483 patterns were selected.

In 2022, the eSup cut-off values were 0.271 for ESBL-EC isolates and 0.034 for non-ESBL-EC, meaning that a pattern was selected if it appeared in at least 27.1% of ESBL-EC or 3.4% of non-ESBL-EC. This value was relatively stable over time for both ESBL-EC (range: 23.6%–27.1%, *P*-value = 0.22, Mann-Kendall test) and non-ESBL-EC (range: 2.9%–3.4%, *P*-value = 0.13, Mann-Kendall test).

The cLift cut-off value for ESBL-EC and non-ESBL-EC was, respectively, 1.058 and 1.041 in 2022, meaning that a pattern was selected if it was 5.8% (respectively 4.1%) more frequent than expected by chance under the assumption of resistance independence in ESBL-EC (respectively non-ESBL-EC). These cut-offs were again relatively stable over time for both ESBL-EC (*P*-value = 0.46, Mann-Kendall test) and non-ESBL-EC (*P*-value = 0.09, Mann-Kendall test). Overall, this pruning step selected respectively 1,770 (1.43% of patterns) and 93 (2.06%) patterns for ESBL-EC and non-ESBL-EC in 2022. The number of selected patterns had an increasing trend from 2018 to 2022 for both ESBL-EC (*P*-value = 0.03, Pearson test) and non-ESBL-EC (*P*-value = 0.05, Pearson test).

### Multiresistance pattern analyses

#### Resistance association networks have a higher density for ESBL-EC

**Fig 3:**
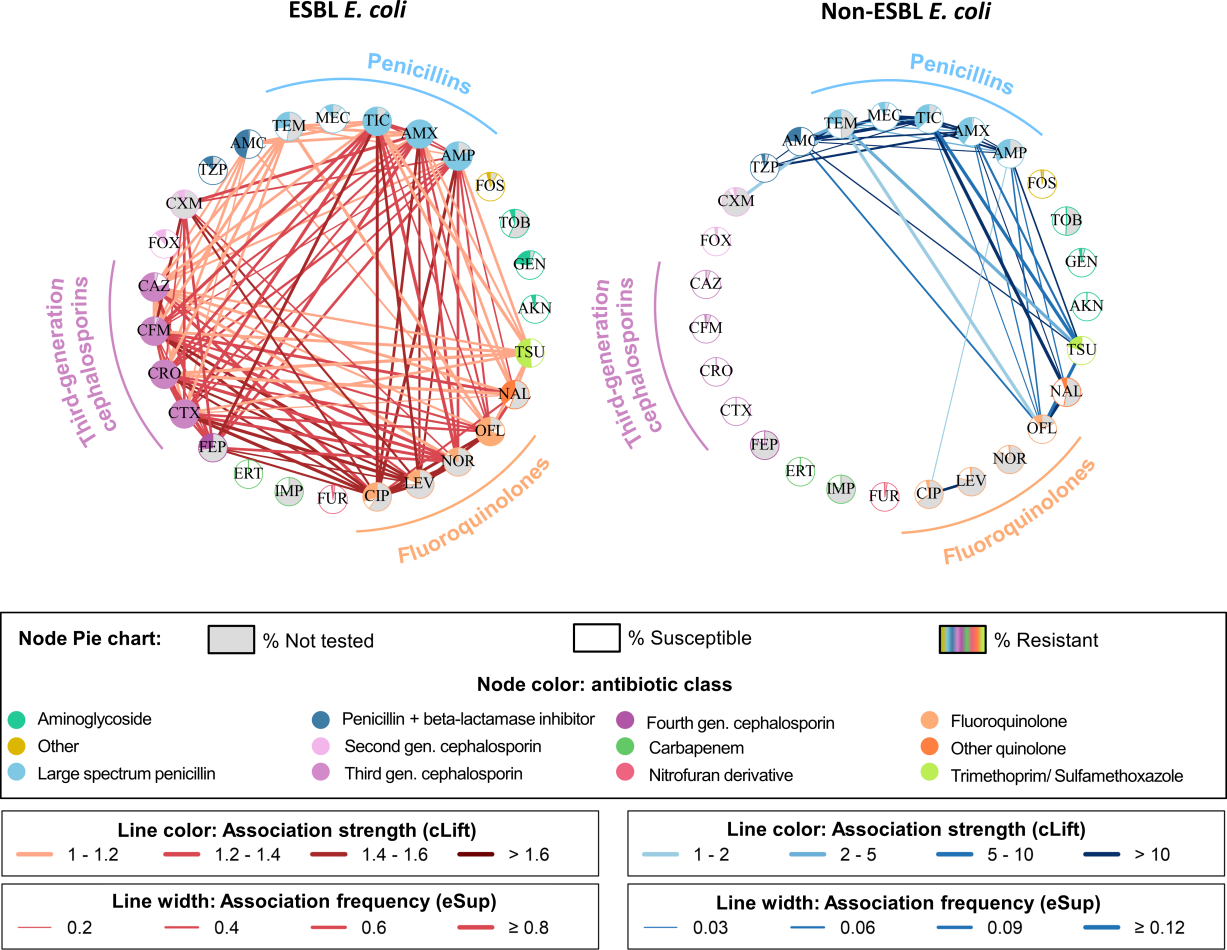
Networks generated from ESBL-EC and non-ESBL-EC in 2022. Patterns were decomposed into nodes (antibiotics) and edges (resistance associations). Node color indicates the antibiotic class. Each node includes a pie chart: Gray represents the proportion of isolates not tested for the antibiotic, white indicates susceptible isolates, and color shows the proportion of resistant isolates. Edges are darker with high cLift values, indicating that the resistance associations occur more frequently than expected under the hypothesis of independence of resistance traits. Edge width increases with high eSup values, reflecting the frequency of resistance associations. See Table S2 for a complete list of antibiotics with their full names.

We decomposed the selected MDR patterns into pairwise associations and built resistance association networks, where nodes represent antibiotics and edges represent resistance associations between two antibiotics. Resistance association networks were much denser in ESBL-EC than non-ESBL-EC, with densities of, respectively, 0.301 and 0.100 in 2022 (Fig. 3). As expected, resistance associations in ESBL-EC involved mainly penicillins, third-generation cephalosporins, and fluoroquinolones. In non-ESBL-EC, strong resistance associations were found between penicillins, combinations of penicillin and beta-lactamase inhibitor, and quinolones. Regarding first-line antibiotics, almost no significant association involving nitrofurantoin, fosfomycin, mecillinam, or any aminoglycoside was found in both ESBL-EC and non-ESBL-EC. However, trimethoprim/sulfamethoxazole was associated with third-generation cephalosporins in ESBL-EC and with penicillins in non-ESBL-EC.

#### Network density increases over time for both ESBL-EC and non-ESBL-EC

**Fig 4:**
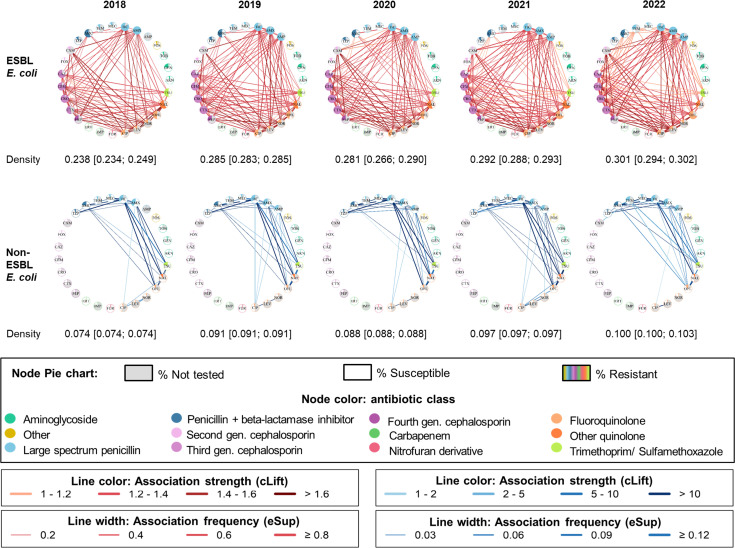
Networks of resistance associations from 2018 to 2022. Median density and 95% CI calculated using: (i) for 2018, 10 bootstrapped data sets; (ii) for 2019 to 2022, 10 subsamples randomly drawn to match the size of the 2018 data set. See Table S2 for a complete list of antibiotics with their full names.

We assessed temporal changes in resistance associations network density, adjusting for increasing data set size by randomly subsampling the 2019 to 2022 data at the size of the 2018 data set. Network density increased over time, from 0.238 (0.234; 0.249) in 2018 to 0.301 (0.294; 0.302) in 2022 for ESBL-EC (*P*-value = 0.06, Pearson test), and from 0.074 (0.074; 0.074) in 2018 to 0.100 (0.100; 0.103) in 2022 for non-ESBL-EC (*P*-value = 0.04, Pearson test; Fig. 4). In addition, we calculated cumulative rule stability (CRS), which quantifies the proportion of shared patterns over time (9, 14) separately for ESBL-EC and non-ESBL-EC from 2018 to 2022. CRS was 0.47 for ESBL-EC and 0.70 for non-ESBL-EC, indicating that resistance patterns were more stable over time in non-ESBL-EC isolates.

#### Network density is higher in individuals 65+ years old

**Fig 5:**
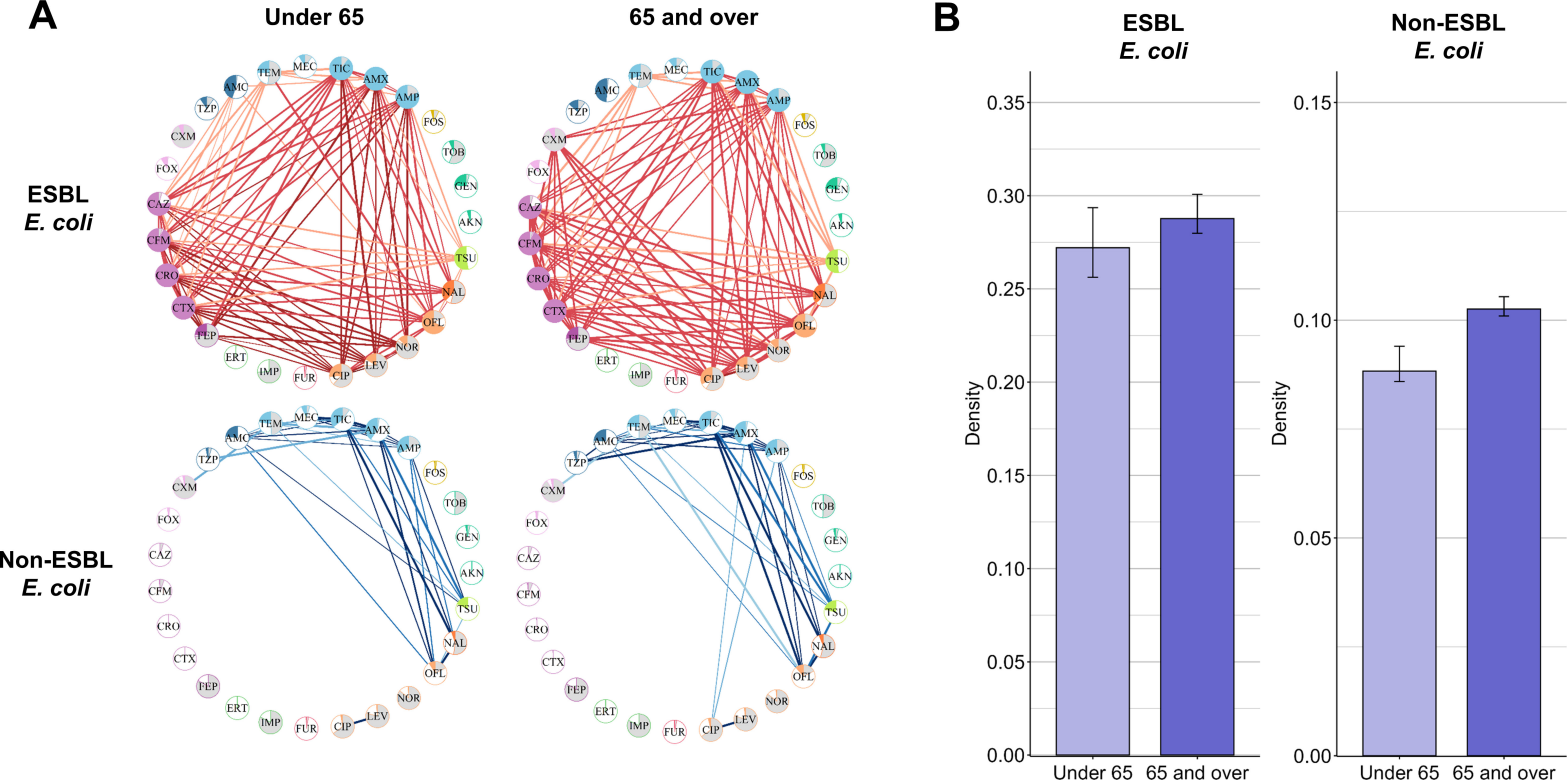
(**A**) Networks of resistance associations for individuals under 65 and individuals 65 and over in 2022. (**B**) Bar plots of median network density for both age groups. Error bars indicate confidence interval: for individuals over 65, calculated using 10 subsamples randomly generated at the size of the data set of individuals under 65; for individuals under 65, calculated using 10 bootstrapped data sets from the complete data set of individuals under 65. See Table S2 for a complete list of antibiotics with their full names.

We also compared networks between individuals under and over 65 years old, again adjusting for different data set size through a subsampling procedure. For non-ESBL-EC, resistance association networks were denser in individuals aged 65 and over than in individuals under 65, with median densities of respectively 0.103 (0.103; 0.105) and 0.088 (0.088; 0.091) in 2022 (Fig. 5B). For ESBL-EC, the median network density was not significantly different between 65+ years old, at 0.289 (0.280; 0.301), and –65 years old, at 0.275 (0.261; 0.294). The ESBL-EC networks of 65+ individuals mainly involved additional associations of cefuroxime with penicillins (amoxicillin and ampicillin), third and fourth generation cephalosporin (cefotaxime and cefepime), and fluoroquinolones (ciprofloxacin, levofloxacin, and norfloxacin; Fig. 5A). We calculated the rule overlap ratio (ROR), which describes the proportion of shared patterns between two datasets, between individuals under 65 and individuals aged 65 and over. In 2022, the median ROR was 0.55 (0.50–0.59) for ESBL-EC and 0.76 (0.75–0.78). Similar results were found when analyzing data from 2018 to 2021 (Fig. S5).

#### Network density is higher in men

**Fig 6:**
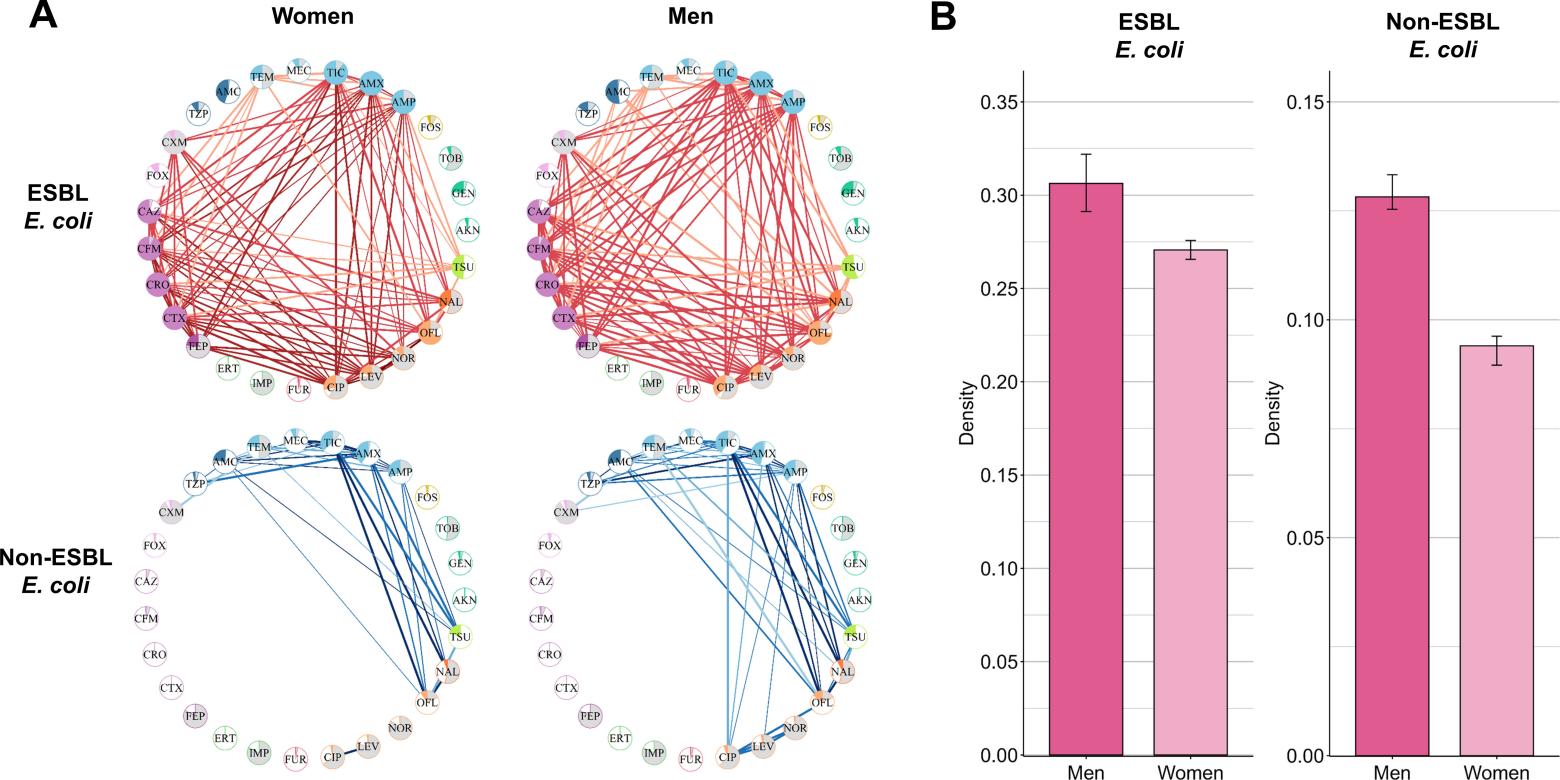
(**A**) Networks of resistance associations for men and women in 2022. (**B**) Bar plots of the median network density for men and women. Error bars indicate confidence intervals: for women, calculated using 10 data sets generated at the size of the men’s data set; for men, calculated using 10 bootstrapped data sets from the complete men’s data set. See Table S2 for a complete list of antibiotics with their full names.

We compared resistance association networks between men and women, again adjusting for different data set size through a subsampling procedure. In 2022 for ESBL-EC, network density was higher in men than in women, with respective medians of 0.305 (0.291; 0.322) and 0.271 (0.266; 0.276; Fig. 6B). The same result was observed in non-ESBL-EC, with median densities of 0.128 (0.125; 0.133) and 0.094 (0.090; 0.096) for men and women, respectively. The ESBL-EC networks of men mainly involved additional associations of cefuroxime with penicillins (amoxicillin and ampicillin), third- and fourth-generation cephalosporins (cefotaxime and cefepime), and fluoroquinolones (ciprofloxacin, levofloxacin, and norfloxacin; Fig. 6A). Similarly to age-specific comparisons, we calculated the ROR between men and women. In 2022, the median ROR between men and women was 0.35 (0.33–0.41) for ESBL-EC and 0.69 (0.67–0.70) for non-ESBL-EC. Similar results were found when analyzing data from 2018 to 2021 (Fig. S6).

#### Sensitivity analysis

To investigate the impact of regional contribution variations on our results (Fig. S1), we conducted analyses on resampled data sets in which the relative contribution of each region was constant over time and proportional to its population (Fig. S7). As in the main analysis, networks reconstructed for ESBL-EC isolates were much denser than for non-ESBL-EC. For ESBL-EC, a significant increase in density was again observed over time, from 0.240 to 0.300 (*P*-value = 0.004, Pearson test), although this was not the case anymore for non-ESBL-EC.

In addition, to evaluate the influence of the arbitrary criterion used for antibiotic selection, we performed the analyses with a stricter criterion requiring each antibiotic to be tested on at least 10% of isolates in each year. The resistance associations identified were consistent with those in the main analysis (Fig. S8).

## DISCUSSION

In this study, we identified resistance associations in community-acquired UTI *E. coli* isolates, using a large French surveillance database. Across all years, and as expected, ESBL-EC showed a higher number of resistance associations, particularly involving penicillins, fluoroquinolones, and third-generation cephalosporins. In both ESBL and non-ESBL-EC, the number of resistance associations increased from 2018 to 2022, and men and individuals aged 65 and over showed more resistance associations across all years.

We compared the observed distribution of the number of resistances per isolate with a simulated data set assuming the hypothesis of mutual independence of resistance traits (10) and found significant differences in non-ESBL-EC. This finding aligns with Chang et al., 2015 (7), which reported that MDR occurred at frequencies inconsistent with the hypothesis of mutual independence of resistance traits for multiple bacteria including *E. coli* (7). It provides further evidence for biological or genetic mechanisms favoring the co-occurrence of multiple resistance traits in *E. coli*. For ESBL-EC, we found no significant difference between the observed and simulated distributions. This indicates that the average number of antibiotics to which an isolate is resistant does not differ from what would be expected under the hypothesis of mutual independence of resistance traits. However, this does not imply that resistance traits are independent in ESBL-EC.

Resistance rates were generally low for first-line antibiotics recommended in France for community-acquired UTIs, such as fosfomycin, pivmecillinam, or nitrofurantoin (15). Analysis of ESBL-EC resistance association networks highlighted major associations between penicillins (± inhibitors), cephalosporins, fluoroquinolones, and cotrimoxazole. This pattern may correspond to CTX-M, the most prevalent ESBL in *E. coli*, as well as TEM or SHV enzymes, which all hydrolyze penicillins and cephalosporins. CTX-M is mostly susceptible to piperacillin-tazobactam (~80%–90%), while this is more variable in TEM/SHV. They are both highly resistant to fluoroquinolones (80%–90%) and moderately resistant (~50%) to cotrimoxazole. Although the mechanisms of resistance to beta-lactams and fluoroquinolones are distinct, fluoroquinolone resistance genes (e.g., qnr or aac[6’]-Ib-cr) are present on mobile genetic elements that also carry beta-lactam resistance genes, which allows multiple resistances to be transmitted jointly (16). Association-set mining may represent an efficient approach to better understand the dynamics of *E. coli* multiresistance patterns and make hypotheses regarding the underlying genetic evolutions, to be confirmed by whole-genome sequencing analysis.

Using association-set mining, we found that the number of resistance associations increased from 2018 to 2022 in both ESBL-EC and non-ESBL-EC. Despite this overall increasing trend, a temporary decrease was observed in 2020, consistent with the observed reduction in MDR *E. coli* during the COVID-19 pandemic in France (17). We explored the impact of spatial resistance epidemiology on our findings through a sensitivity analysis using data sets where the number of isolates from each region was proportional to its population. This approach confirmed the nationwide increase of resistance associations within community-acquired ESBL-EC UTIs. However, in this sensitivity analysis, the increase in non-ESBL-EC disappeared. Consequently, the changes in regional relative contributions may have played a role in the temporal increase of the number of resistance associations we observed in non-ESBL-EC isolates. In particular, the contribution of several densely populated and internationally connected French regions, including Ile-de-France (Paris area), increased over time. Isolates from these regions may present more resistance associations. In addition, we showed this increasing trend over a 5-year period, but these findings should be confirmed over a longer time frame.

During 2018–2022, the global antibiotics consumption in primary care decreased in France from 22.9 to 21.6 defined daily doses per 10,000 inhabitants per day. This decreasing trend in consumption was observed for large-spectrum penicillins, cephalosporins, and quinolones, but not for associations of penicillins, sulfonamides, and trimethoprim, which remained stable. In this context, the increasingly dense MDR patterns we observe among community *E. coli* may be driven not only by antibiotic use but also by other multiple human, animal, and environmental factors (18).

Isolates coming from men and individuals older than 65 had more resistance associations, even after adjusting for different sample sizes. This finding is consistent with multiple studies showing that older individuals are particularly exposed to MDR bacteria, notably because of immunosenescence, frequent multiple chronic comorbidities, and the associated requirement for care. They are more frequently exposed to hospitals and other healthcare facilities, increasing their contact with resistant bacteria (19, 20). In contrast, limited data are available regarding gender-specific differences in MDR, although some studies suggested that men have a higher risk than women of being infected with resistant bacteria (21–23). In our study, men-specific networks had additional associations involving cefuroxime. In hospital settings, cefuroxime is routinely used as surgical prophylaxis to reduce the risk of postoperative infections, particularly in prostate resection, a common procedure in older men (24). Moreover, UTIs in men are more commonly associated with structural abnormalities like renal stones and malignancies, which can complicate the treatment, lead to increased antibiotic use, and contribute to the development of resistance (25). This could partially explain the additional associations. To explore a potential gender-by-age interaction, we performed separate analyses for men over 65, men under 65, women over 65, and women under 65 (Fig. S9). For ESBL-EC, network density was significantly lower in women under 65 compared to the other groups. This may be explained by the high prevalence of uncomplicated cystitis in this population, which typically involves fewer resistance traits. For non-ESBL-EC, men over 65 exhibited significantly higher network density than the other groups. This group’s higher level of healthcare exposure likely increases their risk of acquiring further resistance traits.

Our study is subject to certain limitations that should be taken into account.

First, the size of the PRIMO data sets generally increased every year, notably due to the increase in the scope of the PRIMO mission. However, the 2020 data set was slightly smaller than the 2019 one (450,135 vs 450,820 isolates). This could be partially explained by the COVID-19 pandemic, which disrupted healthcare systems and microbiological surveillance practices in 2020. Community clinical laboratories prioritized COVID-19 management, potentially reducing their capacity to collect and record AST (26). While network size is known to impact its density (27), in our analyses, we accounted for varying sizes of compared data sets by randomly drawing subsamples at the size of the smallest data set.

Second, the PRIMO surveillance system included isolates collected in community clinical laboratories. Although these laboratories process exclusively outpatient samples, excluding samples from hospital inpatients and nursing home residents, a small number of isolates may come from individuals who were recently hospitalized and could have acquired the pathogen in that setting. Therefore, we cannot be entirely certain that the data were representative of the total community-acquired *E. coli* UTI in France between 2018 and 2022.

Third, in the absence of definitive UTI clinical diagnoses, we assumed that urine samples were taken in the context of a strong UTI suspicion. A small number of isolates may have come from asymptomatic bacteriuria; however, as all isolates came from community laboratories, it is very likely that these urine samples were collected for diagnostic purposes from patients with clinical symptoms suggestive of a UTI.

Fourth, in the PRIMO database, some antibiotics were tested on a very small number of isolates. To minimize bias, we included only antibiotics tested on at least 10% of isolates across all data sets and excluded antibiotics rarely used in routine. We found stable results for most antibiotics over time. However, this limitation may explain the changing results regarding ampicillin from 2018 to 2019. In 2018, ampicillin was tested on only 2% of isolates, which likely prevented the algorithm from identifying significant associations involving this antibiotic. By 2019, ampicillin was tested on more isolates, allowing the algorithm to detect such associations. We did a sensitivity analysis using a stricter criterion to select antibiotics, which gave results consistent with those of the main analysis. Moreover, the antibiotics tested were determined by the laboratories and might not align with hospital practices, while consistent with practices in outpatient care. Another potential bias is that rarely tested antibiotics in our data might have been tested only on highly resistant isolates. However, a large majority of participating clinical laboratories (90.5%) performed first-line AST in liquid medium, and the frequency of antibiotics tested in our study was consistent with the frequency at which these antibiotics were found in used AST panels, strongly suggesting the absence of bias.

Fifth, we adopted a microbiological perspective and grouped isolates categorized as “Susceptible to increased exposure” with those categorized as “Resistant,” as they show at least partial resistance mechanisms. The “Susceptible to increased exposure” classification can result from various mechanisms, such as hyperproduction of chromosomally encoded beta-lactamase and outer membrane proteins, which confer partial resistance to cefuroxime (28). A clinically oriented analysis might have led to different conclusions.

Sixth, our results are valid for *E. coli* isolates only, and not for other Enterobacterales, potentially limiting their generalizability. We chose to focus the study on *E. coli* as it represented 85.0% of all isolates in the data set.

Seventh, the *Apriori* algorithm is a highly efficient method supported by multiple studies (9–11). However, combining multiple data mining approaches, as recently suggested (11), could have enhanced our understanding of MDR. In addition, *Apriori* generates numerous patterns, requiring careful pruning with appropriate quality measures and cut-off values. While many studies rely solely on support (29), it would have favored frequent resistance patterns, such as those involving amoxicillin, while overlooking rarer but significant associations. We therefore chose lift as a robust complementary metric (9); however, numerous other metrics or metric combinations could be proposed (30, 31), potentially yielding slightly different results. Missing data may also have influenced *Apriori* results by altering resistance prevalence distributions and associations (32), although this was addressed by the use of adapted quality measures (eSup and cLift) (33). Moreover, as *Apriori* returns numerous patterns, we applied initial support thresholds of 0.001 for non-ESBL-EC and 0.01 for ESBL-EC, the latter being stricter due to computational limitations. This may have underestimated network density for ESBL-EC. To evaluate this, we repeated the analyses from 2018 to 2022 with an initial support threshold of 0.001 for ESBL-EC. The resulting networks were much denser, leading to a loss of statistical significance of the density increase over time (*P* = 0.14, Pearson test), possibly reflecting a saturation of network connections (Fig. S10). Finally, cut-off values also influence pattern selection (29). Our percentile-based approach led us to exclude patterns with a lift below 1, which could have highlighted MDR patterns occurring less frequently than expected.

Despite these limitations, our study provided a novel and detailed analysis of multiresistance patterns in community-acquired *E. coli* UTI collected from a French national surveillance system. We explored the temporal evolution of resistance associations, gender-specific and age-specific differences, which to our knowledge, had not been previously analyzed. Our findings confirm that this method is effective for identifying resistance associations in antibiotic resistance surveillance data. With further research, this work could provide insights for antibiotic stewardship strategies in alignment with known resistance associations in community-acquired *E. coli* UTIs. In the context of rising antibiotic resistance, optimizing the use of current medications is crucial, as few new antibiotics have been developed in the past two decades (34).

Future research could use other machine learning approaches to further analyze the PRIMO data sets and get a deeper understanding of resistance associations. Moreover, it would be interesting to examine the evolution of resistance associations in future years. In addition, extending our approach to other pathogens beyond *E. coli* could offer a broader perspective on multiresistance dynamics. Finally, future work could investigate the potential linkage between these phenotypic resistance associations and genetic co-resistance mechanisms, depending on the availability of genotypic data.

## MATERIALS AND METHODS

### Data source

This study used retrospective data collected via PRIMO, a French nationwide surveillance system of clinical laboratories participating on a voluntary basis (742 laboratories in 2018, 1773 laboratories in 2022) (12). Data included AST results, patient’s age, gender, and administrative region. We restricted the analyses to AST collected from *E. coli* urine samples in community laboratories between 1 January 2018 and 31 December 2022, excluding nursing homes. While no definitive clinical diagnosis was available, we assumed that urine samples were taken in the context of a strong UTI suspicion.

AST and ESBL testing were carried out and assessed according to the recommendations of the AST committee of the French Microbiology Society (35). The presence of ESBL was confirmed by either the double-disk synergy test (44% of clinical laboratories) or the combination disk test (39%). The remaining laboratories used alternative methods, such as colorimetry, immunochromatography, or a combination of two methods (36). Each isolate was tested against 3–35 antibiotics and categorized in a Susceptible, Susceptible to increased exposure, or Resistant format according to the European Committee on Antimicrobial Susceptibility Testing recommended breakpoints, updated every year (Table S1). For this analysis, Susceptible to increased exposure and Resistant were grouped as Resistant. We excluded antibiotics that were tested on less than 10% of isolates on average across all years and antibiotics rarely used in routine. This process resulted in the inclusion of 27 antibiotics (Table S2). As the EUCAST breakpoint defining “Susceptible” isolates did not change over the study period for any of these antibiotics, our findings were not affected by breakpoint changes. Carbapenemase-producing isolates were excluded from the analysis as their low frequency of testing did not allow for a robust analysis of their specific multiresistance patterns. To avoid duplicates, when multiple isolates with the same susceptibility pattern were collected from the same individual (same date of birth and sex) within a single clinical laboratory, only the first isolate in the data set was included.

### Data stratification

We classified isolates into two phenotype categories: ESBL-EC, either alone or combined with another phenotype, and non-ESBL-EC. Table S3 presents the frequency of each phenotype combined with ESBL production. Isolates with other combined phenotypes represented less than 0.01% of all ESBL-EC across all years. We conducted analyses separately for each year and phenotype data set. To explore potential gender- and age-based differences in multiresistance patterns, we also conducted stratified analyses by sex and separate analyses for individuals aged under and over 65.

### Testing the independence of individual resistances

We first assessed the independence of individual resistances (10). We simulated 100 data sets under the hypothesis that all antibiotic resistance traits are mutually independent, which matched the observed data sets in size, resistance prevalence, and positions of missing AST results. Resistance traits were generated as independent binomial random variables (*n* = number of isolates tested; *P* = resistance prevalence). To evaluate the likelihood of the observed MDR prevalence under the hypothesis of mutual independence of all antibiotic resistance traits, we compared the distribution of resistance counts per isolate between observed and simulated data using a Kolmogorov-Smirnov test.

### Multiresistance patterns identification

To identify MDR patterns from our data sets, we used association-set mining with the *Apriori* algorithm, as previously proposed by Cazer et al. (10, 13). Briefly, we first explored the data sets to select all patterns present with a given frequency, setting the required initial minimum frequency at 0.01 for ESBL-EC and 0.001 for non-ESBL-EC. In a second step, we selected patterns for which two important metrics, eSup and cLift, were significantly higher than expected by chance. To do this, we determined the 95th percentiles of eSup and cLift under the resistance independence hypothesis using the previously described 100 data sets simulated under the hypothesis of mutual independence of all antibiotic resistance traits, and pruned all patterns with eSup and cLift below these cut-off values. eSup measures the pattern frequency within the data set, while cLift assesses the frequency of a pattern relative to its expected frequency under the assumption that resistance to antibiotics within the pattern is independent. More details on multiresistance pattern identification and accounting for missing data are provided in Text S1.

We performed a trend test to determine if there was an increasing or decreasing trend in the cLift and eSup cut-off values over the years, as well as in the number of patterns initially identified by *Apriori* and those selected after the pruning step. The decision to use either the Pearson test or the Mann-Kendall test was made by fitting a linear model to the data and evaluating the residuals for normality and homoscedasticity using the Shapiro-Wilk and Breusch-Pagan tests, respectively. When at least one of these assumptions was violated, we opted for the Mann-Kendall test instead of the Pearson test.

### Construction and analysis of resistance association networks

We decomposed the MDR patterns remaining after pruning into pairwise associations. For example, a pattern {A,B,C} was decomposed into three pairwise associations: {A,B}, {B,C}, and {A,C}. We built resistance association networks from these associations, where nodes represent antibiotics, and edges represent resistance associations between two antibiotics, following the method and adapting the publicly available code provided by Cazer et al. (10) (available at https://doi.org/10.5281/zenodo.3887072).

For each reconstructed network, we computed network density. Confidence intervals were estimated using: (i) for 2018, 10 data sets generated by a bootstrap procedure of the original data set, (ii) for 2019 to 2022, 10 random subsamples of the original data sets, each created to match the size of the 2018 data set. We calculated the 95% confidence intervals from the 2.5th and the 97.5th percentiles of the results. Time changes in network density were assessed using these constant-size yearly samples and a Pearson trend test.

Networks were also compared between men and women and individuals under or over 65 years old. Similar to between-year comparisons, we generated 10 random subsamples of the women’s (respectively over-65) data set to match the size of the men’s (respectively under-65) data set and computed 95% confidence intervals from either bootstrapped data or these subsamples.

We compared MDR patterns following the approach of Cazer et al., 2019 (9), using ROR and CRS. ROR (14) is calculated between two datasets and measures the proportion of shared patterns relative to the total number of patterns in both data sets. CRS (14) averages the ROR over time for consecutive rule sets.

### Sensitivity analyses

The regional contributions to the national data set varied over the years. To investigate the impact of these variations on our results, we created a data set where the number of isolates from each region was proportional to its population (details in Text S2) and replicated our analyses using this data set.

In addition, antibiotics were selected based on an arbitrary criterion requiring that each be tested on at least 10% of isolates on average across all data sets. To ensure the reliability of the results, we performed the analyses using a more stringent criterion for antibiotic selection, requiring that the antibiotics be tested on at least 10% of isolates in each year. All analyses were performed in R 4.3.2, notably using the package “arules” for association-set mining (37).

## Data Availability

All codes used in the analysis are available in the following GitHub repository: https://github.com/elisehodbert/exploring_MDR_patterns.

## AFTER EPUB

*[This article was published on 31 October 2025 with an error in the author affiliations. The affiliation was corrected in the current version, posted on 7 November 2025.]*

## SUPPLEMENTAL MATERIAL

Supplemental material aac.00422-25-s0001.docx Tables S1 to S3; Fig. S1 to S10.

ASM does not own the copyrights to Supplemental Material that may be linked to, or accessed through, an article. The authors have granted ASM a non-exclusive, world-wide license to publish the Supplemental Material files. Please contact the corresponding author directly for reuse.
